# Applying Data Envelopment Analysis to Preventive Medicine: A Novel Method for Constructing a Personalized Risk Model of Obesity

**DOI:** 10.1371/journal.pone.0126443

**Published:** 2015-05-14

**Authors:** Hiroto Narimatsu, Yoshinori Nakata, Sho Nakamura, Hidenori Sato, Ri Sho, Katsumi Otani, Ryo Kawasaki, Isao Kubota, Yoshiyuki Ueno, Takeo Kato, Hidetoshi Yamashita, Akira Fukao, Takamasa Kayama

**Affiliations:** 1 Department of Public Health, Yamagata University Graduate School of Medicine, Yamagata, Japan; 2 Department of Healthcare Management, Teikyo University Graduate School of Public Health, Tokyo, Japan; 3 Department of Clinical Oncology, Yamagata University Faculty of Medicine, Yamagata, Japan; 4 Genome Informatics Unit, Institute for Promotion of Medical Science Research, Yamagata University Faculty of Medicine, Yamagata, Japan; 5 First Department of Internal Medicine, Yamagata University Faculty of Medicine, Yamagata, Japan; 6 Second Department of Internal Medicine, Yamagata University Faculty of Medicine, Yamagata, Japan; 7 Third Department of Internal Medicine, Yamagata University Faculty of Medicine, Yamagata, Japan; 8 Department of Ophthalmology and Visual Sciences, Yamagata University Faculty of Medicine, Yamagata, Japan; 9 Department of Neurosurgery, Yamagata University Faculty of Medicine, Yamagata, Japan; Innsbruck Medical University, AUSTRIA

## Abstract

Data envelopment analysis (DEA) is a method of operations research that has not yet been applied in the field of obesity research. However, DEA might be used to evaluate individuals’ susceptibility to obesity, which could help establish effective risk models for the onset of obesity. Therefore, we conducted this study to evaluate the feasibility of applying DEA to predict obesity, by calculating efficiency scores and evaluating the usefulness of risk models. In this study, we evaluated data from the Takahata study, which was a population-based cohort study (with a follow-up study) of Japanese people who are >40 years old. For our analysis, we used the input-oriented Charnes-Cooper-Rhodes model of DEA, and defined the decision-making units (DMUs) as individual subjects. The inputs were defined as (1) exercise (measured as calories expended) and (2) the inverse of food intake (measured as calories ingested). The output was defined as the inverse of body mass index (BMI). Using the β coefficients for the participants’ single nucleotide polymorphisms, we then calculated their genetic predisposition score (GPS). Both efficiency scores and GPS were available for 1,620 participants from the baseline survey, and for 708 participants from the follow-up survey. To compare the strengths of the associations, we used models of multiple linear regressions. To evaluate the effects of genetic factors and efficiency score on body mass index (BMI), we used multiple linear regression analysis, with BMI as the dependent variable, GPS and efficiency scores as the explanatory variables, and several demographic controls, including age and sex. Our results indicated that all factors were statistically significant (p < 0.05), with an adjusted R^2^ value of 0.66. Therefore, it is possible to use DEA to predict environmentally driven obesity, and thus to establish a well-fitted model for risk of obesity.

## Introduction

Personalized medicine considers the patient’s genetic characteristics and history of exposure to environmental factors, and is expected to comprise the next generation of therapy. In this context, personalized medicine has the potential to provide preventive and curative interventions that are personalized for each individual, and is anticipated to reduce the incidence of disease. Furthermore, the completion of the Human Genome Project has paved the way for significant developments in the field of personalized medicine [1]. However, genomic information alone is not sufficient for providing personalized medicine, because the development of non-communicable diseases is a multifactorial process that also involves environmental influences, lifestyle choices, and genetic influences on the individual’s susceptibility. Therefore, population-based prospective cohort studies that incorporate genomic measurements have recently been recommended for determining the risk factors and etiologies of chronic diseases, while also accounting for gene-environment interactions [2–4]. However, these studies are expensive to conduct, and typically require 20–30 years to gather the relevant information [2–4]. Therefore, these challenges are major obstacles in establishing personalized preventative medicine, as the development of predictive models for disease susceptibility are essential to establishing personalized preventative treatments, and these models must incorporate both genetic and environmental factors. Historically, multivariate regression models have commonly been used to identify disease risk factors, although more flexible methods (such as machine learning) are currently used to predict the onset of disease [5].

In this context, data envelopment analysis (DEA) is a measure of efficiency that considers multiple inputs and outputs, and can be used to evaluate outputs while controlling for the inputs [6–8]. The DEA process calculates efficiency scores (a measure of relative efficiency) within a given sample of decision making units (DMUs) [9,10]. A completely efficient subgroup will provide an efficiency score of 1, while a DMU that achieves a score of <1 is considered inefficient, and these scores can be used to calculate an individual’s disease susceptibility. For example, if a person who has a high body mass index (BMI), consumes too much food, and never exercises, that person may be obese due to his/her environment. In contrast, if a person with a high BMI eats appropriately and exercises a regularly, that person’s obesity may be hereditary, which is independent of his/her environment. Therefore, by calculating and comparing individuals’ efficiency scores via DEA, we can evaluate individuals’ susceptibility to obesity. In that model, the inputs would be the individual’s caloric intake and energy expenditure, and the efficiency score risk model would be highly fitted for the onset of obesity, due to the use of BMI as the objective variable (output).

In the field of healthcare, DEA has already been adopted in hospital management [10,11], and one recent study has used DEA to evaluate the efficiency of physical activity programs for elderly women [12]. In that study, the inputs were defined as the amount of time spent performing strength, flexibility, and aerobic exercises, and the outputs were defined as the levels of strength, flexibility, static balance, dynamic balance, and maximal oxygen consumption that were verified at the end of the physical activity program. The authors found that DEA facilitated the assessment of the exercise program using the time spent performing physical activity, and subsequently concluded that DEA has promising applications in preventing fitness-related conditions. However, that study’s findings have limited application in clinical or preventive practice.

Therefore, in the present study, we evaluated DEA in the field of preventive medicine, and investigated its feasibility using clinical and genetic data from a large prospective cohort of a genome-wide association study (GWAS). Using this information, we hoped to estimate personal susceptibility to obesity, using the efficiency scores for the DMUs (individual subjects), and to evaluate the feasibility of applying DEA in the field of preventative medicine.

## Methods

### DEA analysis

In this study, we employed the input-oriented Charnes-Cooper-Rhodes model of DEA, which is a constant returns-to-scale model that is particularly relevant, given its ability to include multiple inputs and outputs without requiring an a priori function specification [13]. In this context, a DMU is defined as the entity that is responsible for converting the inputs into outputs [14]; therefore, we defined the DMUs as individual subjects. The inputs were defined as (1) total physical expenditure (measured as calories expended) and (2) the inverse of food intake (measured as calories ingested). In DEA, efficiency is defined as high if the input is minimized when the outputs are held constant, or if the output is maximized when the inputs are held constant. Therefore, to fit this definition, we used the inverse of BMI and of caloric intake as the outputs for our models, as subjects can only directly control the inputs (e.g., exercise and food intake), and cannot directly control the output (BMI) [15].

Each subject’s efficiency score was calculated using DEA-Solver-Pro Software (Saitech, Inc., Tokyo, Japan) [6]. In that model, the efficiency scores range between 0 and 1, and the individuals who are most efficient at burning calories are assigned an efficiency score of 1 [16].

### Genotyping and imputation

Whole blood samples were used to obtain the subjects’ DNA samples, and the single nucleotide polymorphisms (SNPs) were genotyped using an Infinium 660W BeadChip assay (Illumina, San Diego, CA, USA). For our analyses, SNPs with a minor allele frequency of <0.5% and a call rate of <95% were excluded. After confirming the quality of the genotyped SNP data, we performed genotype imputation using the MACH-Admix program [17], using a data set from the 1000 Genomes Project (194 ASN, 68 CHB, 25 CHS, 84 JPT, and 17 MXL, released in August 2010).

### Genetic predisposition score

The genetic predisposition score (GPS) was calculated using the β coefficients of the SNPs, via a previously reported weighting method [18]. In this method, the GPS is calculated by multiplying the number of effect alleles (0, 1, or 2) at each locus by the *β* coefficient of that SNP (as obtained from the GWAS), dividing by the maximum allowable sum of the *β* coefficients, and then multiplying by twice the number of alleles. Higher scores indicate a greater genetic predisposition to obesity. The GPSs from the β coefficients of 29 SNPs from East Asian subjects were used to conduct these analyses [19,20].

### Statistical analysis

The statistical analyses for this study were conducted during September 2014. The statistical analyses were performed using R software (version 3.1.1, R Foundation for Statistical Computing, Vienna, Austria), and differences with a p-value of <0.05 were considered statistically significant. Pearson’s correlation coefficients were calculated to evaluate the relationships between the efficiency scores, GPS, and BMI. Robust multiple linear regression analyses were also conducted to evaluate the effect of obesity risk factors, using the “robustbase” function in the R software. Five multiple linear regression models were used to evaluate the coefficients for BMI at baseline or yearly changes in BMI (the dependent valuables). Model 1 evaluated the effect of environmental factors (energy expenditure and energy intake) as the explanatory variables, while Model 2 evaluated the effect of the efficiency score. Model 3 evaluated the effect of genetic factors using the GPS, Model 4 evaluated the effect of genetic and environmental factors (energy expenditure, energy intake, and GPS), and Model 5 used the GPS and efficiency scores to compare the effect of genetic factors to that of the efficiency score. All models were adjusted for age and sex.

### Data from the cohort study

The Takahata study is a population-based cohort study (with a subsequent follow-up study) of Japanese people who were >40 years old, which sought to clarify the risk factors for certain lifestyle-related conditions, such as diabetes and obesity [21–24]. The details of the study design have been reported previously [21–24], and the participants’ physical activity status was calculated as metabolic equivalents, using The Japan Arteriosclerosis Longitudinal Study Physical Activity Questionnaire [25]. In addition, the daily intake of each nutrient was calculated using a brief self-administered diet history questionnaire [26]. The baseline survey of 3,522 participants was conducted from 2004 to 2006, and 1,620 unique DNA samples were extracted from the participants’ whole blood samples, and we were able to calculate the efficiency scores for those 1,620 participants. Among these participants, 1,079 completed the follow-up survey in 2011, at 5–7 years after the baseline survey, and we classified these participants as underweight (<18.5 kg/m^2^), normal (18.5–24.99 kg/m^2^), and overweight (≥25 kg/m^2^) [27]. Written informed consent was obtained from all participants for the original study. The protocols of both the Takahata study and the present study were approved by the Yamagata University Faculty of Medicine ethics committee.

## Results

### Efficiency score and baseline BMI

**Fig 1:**
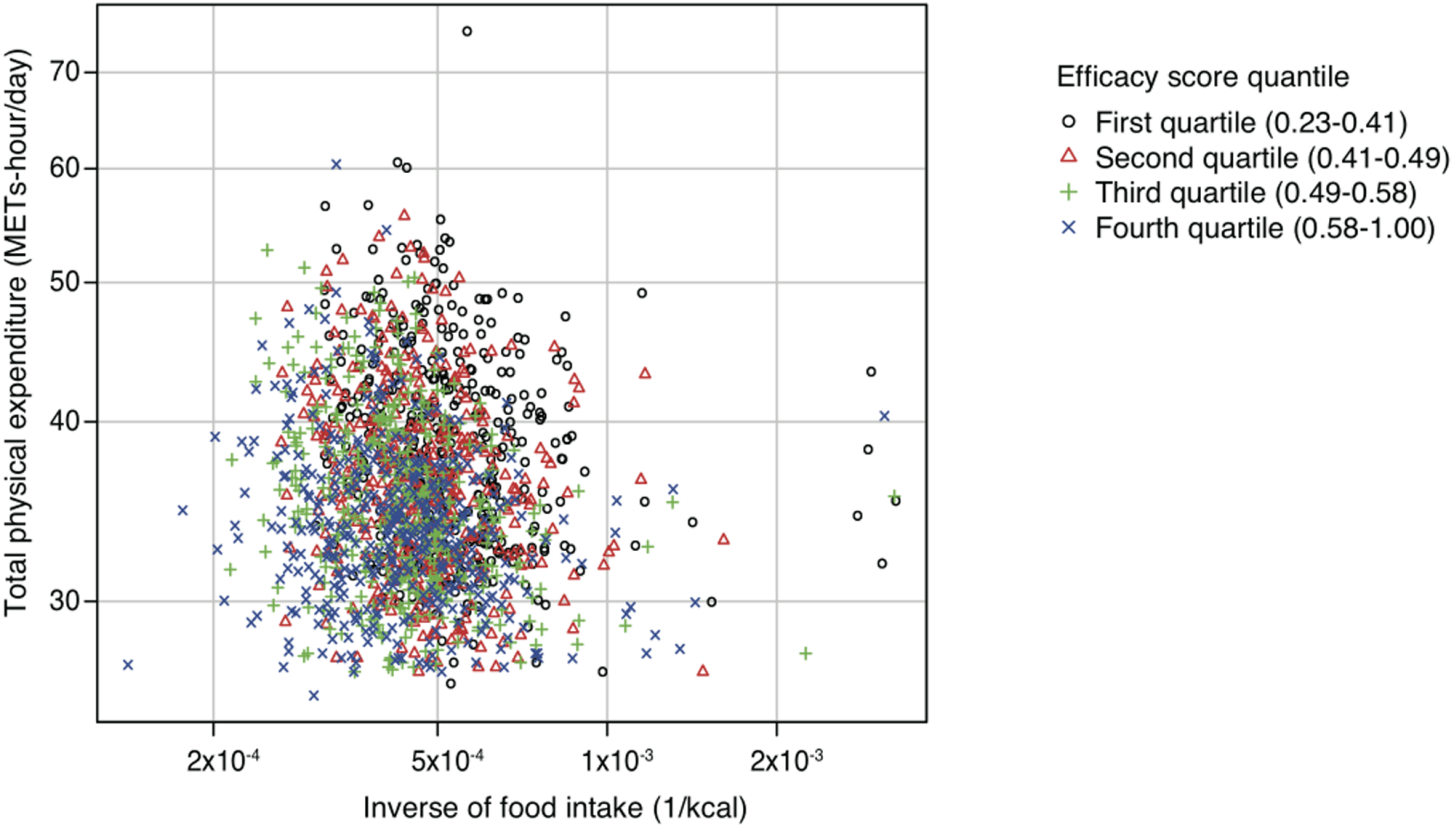
Total physical expenditure and the inverse of food intake according to the efficiency score quartiles. METs, metabolic equivalents.

Among the participants in the Takahata study, both GPS and efficiency scores were available for 1,620 participants (726 men and 894 women). The characteristics of these subjects are shown in Table 1. The median age at the baseline survey was 62 years (range, 40–84 years), and the mean baseline BMI was 23.4 kg/m^2^ (standard deviation [SD], 3.1). The mean efficiency score of the participants was 0.51 (SD, 0.13), and the total physical expenditure and inverse of food intake according to the efficiency score quartiles are shown in Fig 1.

**Table 1:** Participant characteristics (n = 1,620).

Variable	Number
Age, years (median [range], mean [SD])	62 (40–84), 61.3 (10.1)
Sex (men/women)	726/894
Baseline BMI, kg/m^2^ (median [range], mean [SD])	23.2 (15.0–35.5), 23.4 (3.1)
Efficiency score (median [range], mean [SD])	0.49 (0.23–1.00), 0.51 (0.13)
Efficiency score according to age group	
40–49 years	0.49 (0.26–0.91), 0.50 (0.12)
50–59 years	0.47 (0.26–1.00), 0.49 (0.13)
60–69 years	0.49 (0.24–0.99), 0.50 (0.12)
≥70 years	0.54 (0.23–1), 0.55 (0.14)
GPS (median [range], mean [SD])	25.9 (14.5–42.3), 26.1 (3.1)
Total physical expenditure, METs-h/day (median [range], mean [SD])	35.3 (25.8–74.8), 36.2 (5.8)
Food intake, kcal/day (median [range], mean [SD])	2,175 (307–7090), 2,257 (673)
Change in BMI, kg/m^2^/year (median [range], mean [SD])	-0.01 (-0.92–1.14), -0.02 (0.22)

SD: standard deviation; BMI, body mass index; GPS, genomic predisposition score; METs, metabolic equivalents.

**Fig 2:**
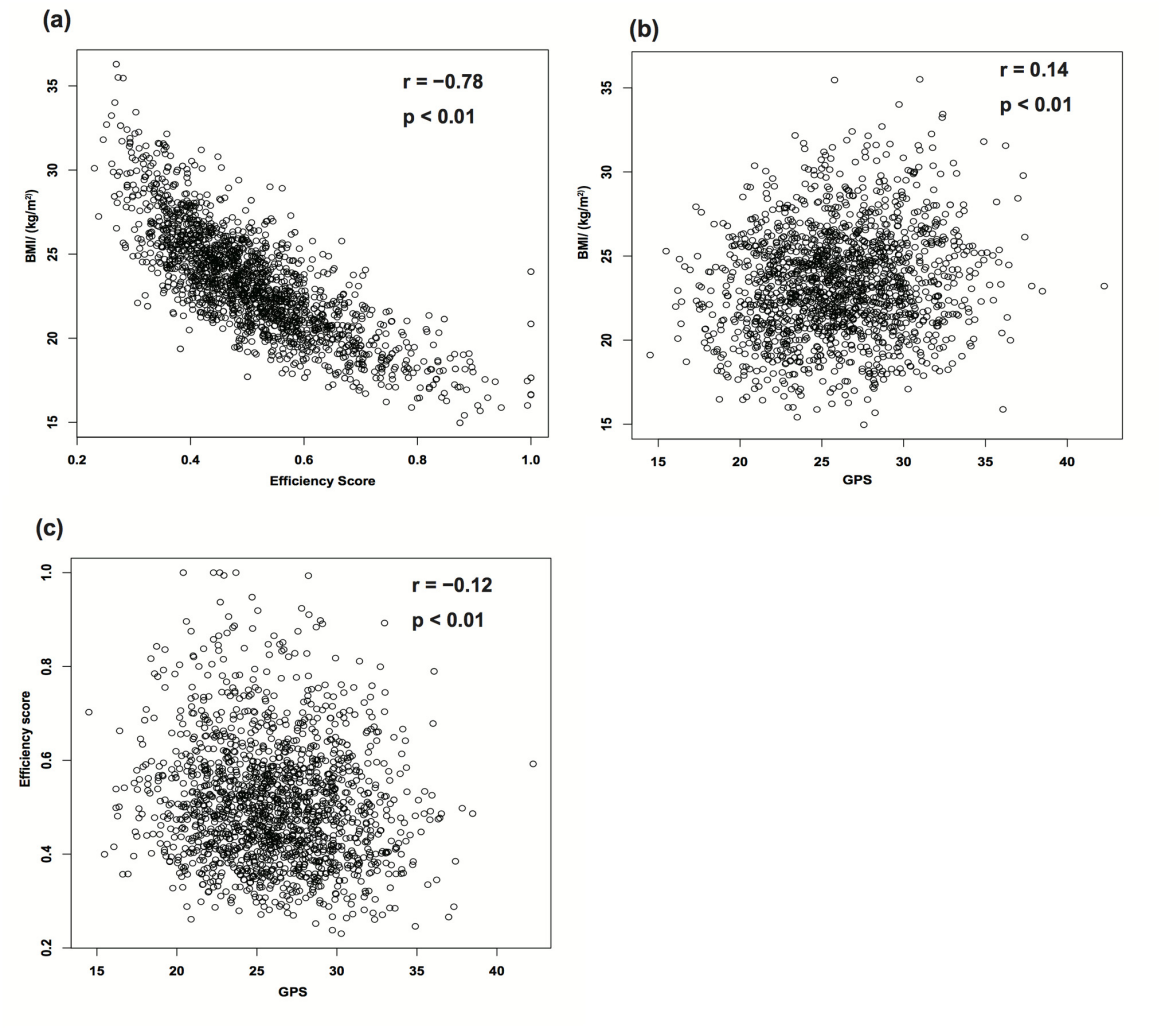
Correlations between clinical variables, genomic predisposition score (GPS), and efficiency score. Correlations are shown for (a) baseline body mass index (BMI) and efficiency score (r = −0.78, p < 0.01), (b) baseline BMI and GPS (r = 0.14, p < 0.01), and (c) efficiency score and GPS (r = −0.12, p < 0.01).

The baseline BMI and efficiency scores were significantly correlated (r = −0.78, p < 0.01) (Fig 2a), as were the baseline BMI and GPS (r = 0.14, p < 0.01) (Fig 2b) and the efficiency scores and GPS (r = −0.12, p < 0.01) (Fig 2c) (Table 2). The results of the subgroup analysis according to age are also shown in Table 2. We then evaluated the coefficients using the five multiple linear regression models, and found that the models that included efficiency score had greater adjusted R^2^ values than those that did not include efficiency score. In Model 5, which used the GPS and efficiency scores to compare the effect of genetic factors with that of efficiency score, the equation for yearly changes in BMI was:

$$\begin{array}{l} Baseline\;BMI\;\left(kg/m^{2}\right)\;=\;28.84\;+\;\left\{0.25\;\times \;Sex\;\left(women\;vs.\;men\right)\right\}\;+\;\{0.05\;\times \;Age\\ \;\;\;\;\;\;\;\;\left(years\right)\}\;+\;\left(0.03\;\times \;GPS\right)\;+\;\left(–19.47\;\times \;efficiency\;score\right)\;\;\; \end{array}$$ (1)

In this formula, all factors were statistically significant (p < 0.05) (Table 3).

**Table 2:** Factors associated with baseline body mass index (n = 1,620).

		Partial coefficient	Standard error†	p-value
Model 1 (adjusted R^2^ = 0.0083)				
Intercept		22.19	0.92	N/A
Sex (women vs. men)		-0.42	0.17	0.01
Age (years)		0.02	0.01	0.03
Energy expenditure (METs-h/day)		0.00	0.01	0.88
Energy intake (kcal/day)		0.0001	0.0001	0.33
Model 2 (adjusted R^2^ = 0.65)				
Intercept		29.76	0.35	N/A
Sex (women vs. men)		0.24	0.10	0.01
Age (years)		0.05	0.0046	<0.01
Efficiency score		-19.57	0.41	<0.01
Model 3 (adjusted R^2^ = 0.025)				
Intercept		19.69	0.75	N/A
Sex (women vs. men)		-0.42	0.16	0.01
Age (years)		0.02	0.01	0.02
GPS		0.10	0.02	<0.01
Model 4 (adjusted R^2^ = 0.024)				
Intercept		19.39	1.07	N/A
Sex (women vs. men)		-0.38	0.16	0.02
Age (years)		0.02	0.01	0.03
Energy expenditure (METs-h/day)		0.0001	0.01	0.99
Energy intake (kcal/day)		0.0001	0.0001	0.31
GPS		0.10	0.02	<0.01
Model 5 (adjusted R^2^ = 0.66)				
Intercept		28.84	0.48	N/A
Sex (women vs. men)		0.25	0.10	0.01
Age (years)		0.05	0.0046	<0.01
GPS		0.03	0.01	0.01
Efficiency score		-19.47	0.41	<0.01
Age <60 years (n = 672) (adjusted R2 = 0.65)	Intercept	30.80	0.94	N/A
	Sex (women vs. men)	-0.02	0.16	0.92
	Age (years)	0.02	0.01	0.09
	GPS	0.03	0.02	0.08
	Efficiency score	-19.78	0.67	<0.01
Age ≥60 years (n = 948) (adjusted R2 = 0.66)	Intercept	27.84	0.83	N/A
	Sex (women vs. men)	0.44	0.12	<0.01
	Age (years)	0.07	0.01	<0.01
	GPS	0.04	0.02	0.02
	Efficiency score	-19.39	0.53	<0.01

N/A, not applicable; GPS, genomic predisposition score; METs, metabolic equivalents.

^†^Robust standard errors are reported.

**Table 3:** Factors associated with yearly change in body mass index (n = 708).

	Partial coefficient	Standard error†	p-value
Model 1 (adjusted R^2^ = 0.032)			
Intercept	0.22	0.09	N/A
Sex (women vs. men)	-0.02	0.02	0.29
Age (years)	-0.004	0.001	<0.01
Energy expenditure (METs-h/day)	0.0004	0.001	0.79
Energy intake (kcal/day)	0.000001	0.00001	0.94
Model 2 (adjusted R^2^ = 0.038)			
Intercept	0.19	0.07	N/A
Sex (women vs. men)	-0.02	0.01	0.16
Age (years)	-0.0043	0.0009	<0.01
Efficiency score	0.14	0.07	0.06
Model 3 (adjusted R^2^ = 0.033)			
Intercept	0.25	0.07	N/A
Sex (women vs. men)	-0.02	0.01	0.25
Age (years)	-0.0041	0.0009	<0.01
GPS	-0.000045	0.0019	0.98
Model 4 (adjusted R^2^ = 0.031)			
Intercept	0.23	0.11	N/A
Sex (women vs. men)	-0.02	0.02	0.29
Age (years)	0.00	0.00	<0.01
Energy expenditure (METs-h/day)	0.0004	0.0015	0.79
Energy intake (kcal/day)	0.000001	0.000013	0.94
GPS	-0.00002	0.0019	0.99
Model 5 (adjusted R^2^ = 0.036)			
Intercept	0.19	0.08	N/A
Sex (women vs. men)	-0.02	0.02	0.16
Age (years)	-0.0043	0.0009	<0.01
GPS	0.0001	0.0019	0.97
Efficiency score	0.14	0.07	0.06

N/A, not applicable; GPS, genomic predisposition score; METs, metabolic equivalents.

^†^Robust standard errors are reported.

### Efficiency score and yearly change in BMI

**Fig 3:**
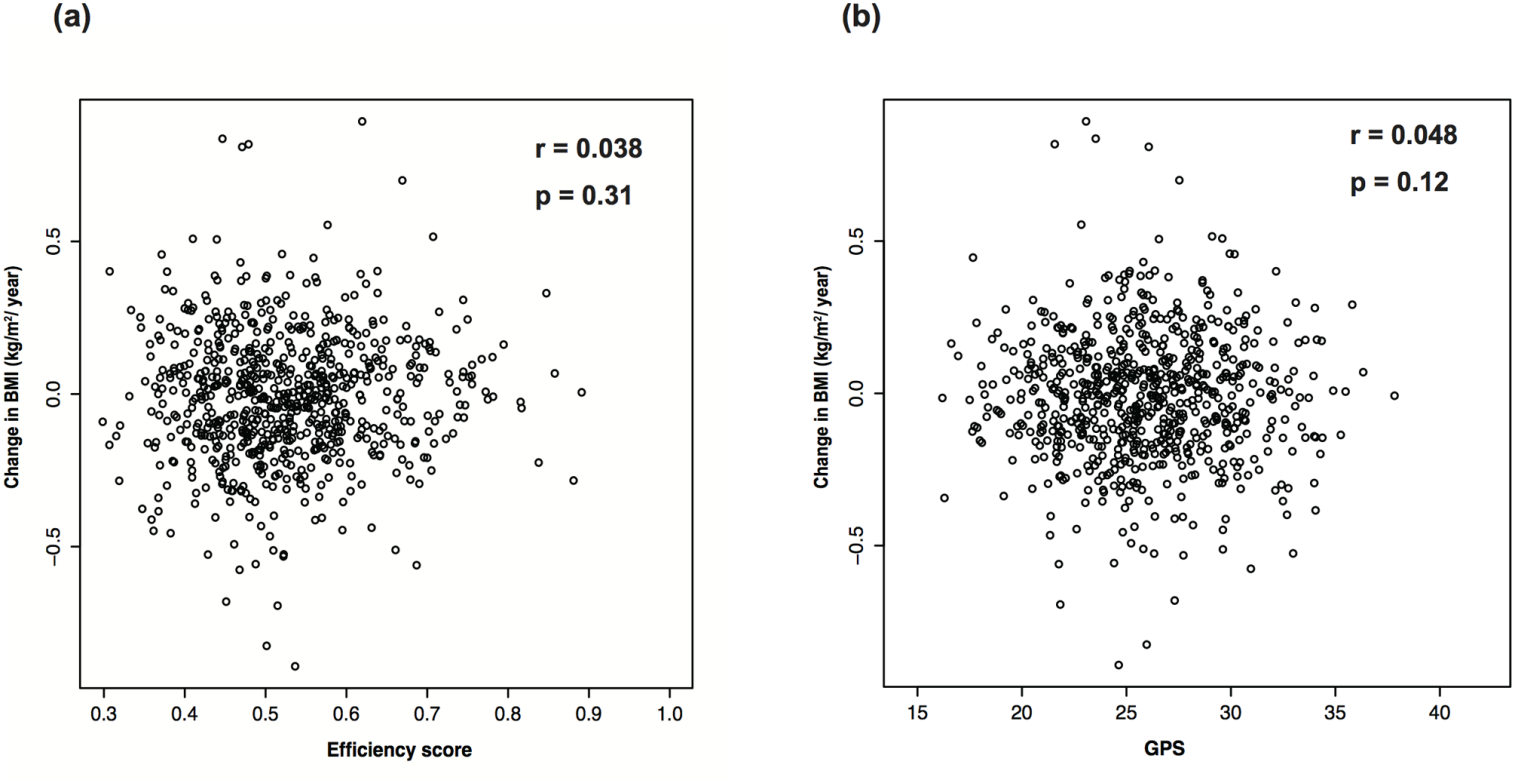
Correlations between changes in body mass index (BMI), genomic predisposition score (GPS), and efficiency score. Correlations are shown for (a) yearly change in BMI and efficiency score (r = −0.012, p = 0.80) and (b) yearly change in BMI and GPS (r = 0.055, p = 0.21).

After excluding underweight and overweight participants, data regarding yearly changes in BMI were available for 708 of the 1,079 subjects who participated in the follow-up survey. The yearly change in BMI was correlated with the efficiency score (r = 0.038, p = 0.31) and the yearly change in BMI and the GPS were also correlated (r = 0.048, p = 0.12) (Fig 3). Furthermore, we evaluated the coefficients for the change in BMI using the five multiple linear regression models, although the R^2^ values for these models were very low (<0.04). In Model 5 (Table 3), we used the GPS and efficiency scores to compare the effect of genetic factors with that of efficiency score, which provided the following equation:

$$\begin{array}{l} Changes\;in\;BMI\;\left(kg/m^{2}/year\right)\;=0.19\;+\;\{–0.02\;\times \;Sex\left(women\;vs.\;men\right)\}\;+\\ \left\{–0.004\;\times \;Age\;\left(years\right)\}\;+\;\left(0.0001\;\times \;GPS\right)\;+\;(0.14\;\times efficiency\;score\right) \end{array}$$ (2)

## Discussion

In this study, we used DEA to estimate individuals’ susceptibility to obesity. First, we compared a regression model that included the GPS with another model that included the efficiency score. Second, we compared the resulting coefficients with the coefficient provided by the model that included both valuables. Based on our results, the efficiency score had a better predictive power when using baseline BMI, compared to GPS, as evidenced by the greater adjusted R2 value and greater partial efficiency value. These findings suggest that DEA is feasible for establishing a model of an individual’s risk of obesity. Similar to the previous study that used DEA to evaluate a physical activity program [12], our data indicate that we can use DEA to predict the efficiency of interventions that are designed to prevent obesity. Therefore, the results of this study highlight the potential applications of DEA in personalized preventative medicine.

Obesity is caused by various factors [19], including environmental (diet and physical activity), genetic, and epigenetic factors [19,28–33]. These genetic factors include common variants (e.g., SNPs), rare variants, and gene-environment interactions [3,34]. In this context, GPS is currently the optimal risk score for evaluating genetic factors, although it can only be measured using a large-scale GWAS, and can only elucidate a subset of the genetic factors. In theory, DEA can calculate an efficiency score that includes all factors, besides the input factors (e.g., food intake and energy expenditure in this study). This theory is strongly supported by our finding that the determination coefficient for the model that included the efficiency score was higher than that for the model that included the GPS. Thus, the efficiency score from DEA is a useful measure for the risk of obesity, given that most genetic factors for the onset of obesity remain unknown. Furthermore, we calculated these efficiency scores using existing clinical data, which is much more cost-effective than personal genomic analyses. Therefore, our methodology can be used as a foundational approach to establishing personalized preventive medicine.

However, there is a major limitation that should be considered when interpreting our findings. For the change in BMI (between the baseline and follow-up surveys), we failed to establish efficiently fitted models, due to the low R^2^ values and low partial efficiencies. This finding may be partially explained by the small value of the absolute change in BMI, which may have been affected by several factors. First, the baseline survey of the Takahata study was conducted when the participants received health check-ups. However, based on the results of that health check-up, the obese participants received preventive medical interventions, such as nutritional guidance. Therefore, it is possible that participants with low efficiency scores had received an intervention, which might have affected their change in BMI. Second, the biological significance of BMI differs according to age, as larger BMI increases are observed in the adolescent period, compared to those observed in adulthood, and BMI peaks at the age of 55 years among men and at the age of 60 years among women [30–33]. However, in the Takahata Study, more than half of the participants were >60 years old, and would likely experience a decreasing trend in their BMI. Thus, the role of the efficiency score should be evaluated among all age groups in future studies, including the adolescent period, as this additional data would facilitate more appropriate use of the efficiency score in preventive medicine. Third, the Charnes-Cooper-Rhodes model is one of many possible models that we could have selected for the present study. However, the present study is the first to examine the application of DEA in predicting obesity, and while model selection is an important consideration in DEA, we were unable to rely on existing studies to justify the model selection. Therefore, we do not reject the possibility that better models may exist for this purpose [15]. Furthermore, we eagerly await future studies in this field, as the better combination of DEA and genetic information would help establish more effective risk models for obesity.

In conclusion, we estimated individuals’ susceptibility to obesity using DEA. Although the results of the present study are preliminary, we are planning large-scale prospective studies to confirm the feasibility and usefulness of DEA in this field. Nevertheless, our findings provide novel foundational insights regarding methods to facilitate personalized preventive medicine.
